# Antimycobacterial Activity and Nitric Oxide Production Inhibition of Antarctic Seaweeds

**DOI:** 10.1021/acsomega.6c01332

**Published:** 2026-09-02

**Authors:** Carolina Rosas Fernández, Thatiana Lopes Biá Ventura Simão, Elena B. Lasunskaia, Ricardo Moreira Borges, Pio Colepicolo, Michelle F. Muzitano, Angélica R. Soares

**Affiliations:** † Grupo de Produtos Naturais de Organismos Aquáticos (GPNOA) − NUPEM, Universidade Federal do Rio de Janeiro, Campus Macaé, Rio de Janeiro, Rio de Janeiro 27965-045, Brazil; ‡ Laboratório de Produtos Bioativos, Instituto de Ciências Farmacêuticas, Universidade Federal do Rio de Janeiro, Campus Macaé, Rio de Janeiro, Rio de Janeiro 27933-378, Brazil; § Laboratório de Biologia Do Reconhecer, Centro de Biociências e Biotecnologia, 28109 Universidade Estadual do Norte Fluminense, Campos dos Goytacazes, Rio de Janeiro 28013-602, Brazil; ∥ Instituto de Pesquisas de Produtos Naturais Walter Mors (IPPN), UFRJ, Rio de Janeiro, Rio de Janeiro 21941-902, Brazil; ⊥ Departamento de Bioquímica, Instituto de Química, 153989 Universidade de São Paulo, São Paulo, São Paulo 05508-000, Brazil; # Laboratório de Produtos Naturais E Bioquímica, Instituto de Pesquisas do Jardim Botânico do Rio de Janeiro, Rio de Janeiro, Rio de Janeiro 22460-030, Brazil

## Abstract

The emergence of increasingly resistant strains of *Mycobacterium tuberculosis* (Mtb) makes the search for new antimycobacterial and host-directed therapies essential to help combat this disease. Antarctica extreme environmental conditions make seaweeds an important source for the discovery of bioactive compounds with the potential to treat various diseases caused by microorganisms. In this study, 29 lipophilic extracts of Antarctic seaweeds were evaluated for their potential as antituberculosis drug sources. The antimycobacterial and immunomodulatory activities (based initially on NO inhibition), as well as the chemical profiles of the algae were studied. Of all the extracts, 21 (72.4%) showed antimycobacterial activity with MIC_50_ values lower than 25 μg/mL against Mtb H37Rv. And, 9 (31%) showed activity also against the hypervirulent strain Mtb M299. Regarding immunomodulation, 27 seaweed extracts (93.1%) inhibited NO production by suppressing iNOS expression with IC_50_ values lower than 25 μg/mL. Six samples of the genera *Desmarestia*, *Sarcopeltis*, *Iridaea*, *Pyropia*, and *Curdiea* with dual activity and a selectivity index higher than ten were selected as promising, with *Pyropia endiviifolia* extract standing out against Mtb H37Rv and Mtb M299 (MIC_50_ 2.1 ± 0.2 and 3.2 ± 0.7 μg/mL, respectively). GC-MS-based Molecular Networking indicated pentadecanoic acid, hexadecanoic acid, and cholesterol as the major known components of the promising samples. Our study demonstrates for the first time the potential of the secondary metabolites from Antarctic seaweeds against Mtb and encourages further investigation to characterize novel antitubercular agents from these extremophilic organisms.

## Graphical abstract

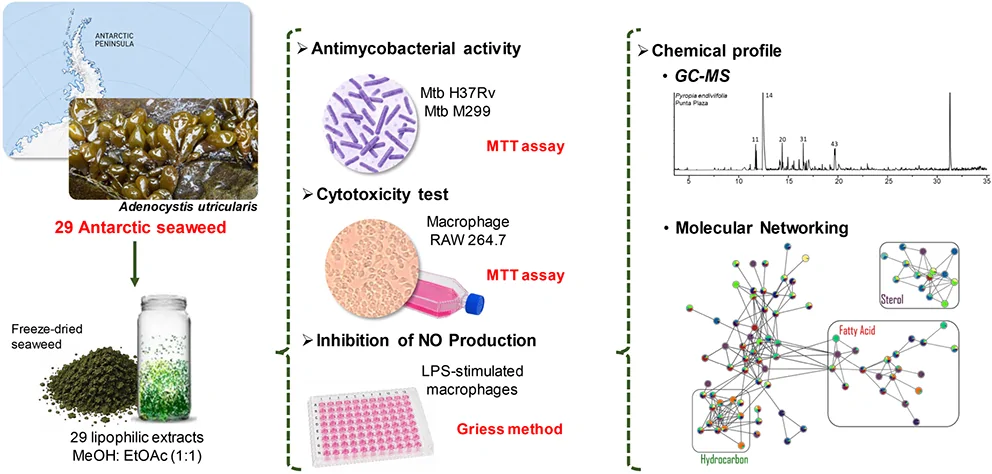

## 1 Introduction

Tuberculosis (TB) is an infectious disease and is the leading cause of death from a single agent. It is estimated that a quarter of the world’s population is infected with *Mycobacterium tuberculosis* (Mtb), and more than ten million new cases of clinical TB and one million deaths are reported annually.^1^ Despite the availability of antituberculosis drugs, the cure rates for MDR-TB and XDR-TB cases are low.^2^

The challenging environmental conditions of the Antarctic continent, associated with limited exposure to sunlight, low temperature conditions, lack of substrate, desiccation, high UV radiation, and high salinity, may induce for adaptation the production of secondary metabolites with potential biological activities.^3–5^ A high proportion of endemic species is described in the Antarctic and are characterized by high temporal and spatial variability.^6^ Regarding Antarctic seaweeds, approximately 40% of all species are endemic.^7,8^ As part of the physiological response to the environment and the restricted climatic and habitat conditions, Antarctic seaweeds have become among the most pristine sources for obtaining compounds with biotechnological potential. The anticancer,^9,10^ antileishmanial,^11^ antifouling, algicidal,^12^ antiviral,^13^ antioxidant,^14^ anti-inflammatory,^15^ and antimicrobial^16^ activities of Antarctic seaweed compounds have already been described. Although no studies have evaluated the antimycobacterial potential of Antarctic seaweeds, several studies have evaluated the crude extracts of seaweeds and their ability to produce compounds against strains of mycobacteria.^17–19^

Knowledge of chemical compounds from Antarctic seaweeds and their pharmacological activities has increased in recent years, and studies on the potential of these organisms for bioprospecting are still limited. In this study, 29 lipophilic extracts from seaweeds collected from different regions of the Antarctic Peninsula were evaluated for their antimycobacterial and immunomodulatory properties. The chemical profiles of the promising extracts were analyzed by using GC-MS and molecular networking.

## 2 Materials and Methods

### 2.1 Study Sites and Organisms

Twenty-nine seaweed samples were collected as a part of the Brazilian Antarctic Program in November–December 2017 from five sampling sites in the South Shetland Islands, Antarctica: Estação Antártica Comandante Ferraz (EACF), Punta Plaza, Yellow Point, and Ipanema on King George Island, and Penguin Islands (Table 1). Seaweeds were collected randomly at low tide in the intertidal zone parallel to the coast. Specimens were carefully washed in cold seawater to remove macroscopic epibiota and sand particles. The material was identified to the species level based on macro- and micromorphological analyses, packed in plastic bags, stored in a freezer at −20 °C, and transported to Brazil. Voucher specimens were deposited in the herbarium of the Instituto de Biodiversidade e Sustentabilidade NUPEM/Federal University of Rio de Janeiro.

**Table 1:** Collection Information and Species Used in This Study

	Species	Collection Site
Chlorophyta		
Monostromataceae	*Monostroma hariotii* Gain	Penguin Island
Ulotrichaceae	*Spongomorpha arcta* (Dillwyn) Kützing	Penguin Island
Ochrophyta		
Adenocystaceae	*Adenocystis utricularis* (Bory) Skottsberg	Penguin Island
	*Adenocystis utricularis*	Punta Plaza
Ascoseiraceae	*Ascoseira mirabilis* Skottsberg	Penguin Island
Desmarestiaceae	*Desmarestia anceps* Montagne	EACF
	*Desmarestia anceps*	Penguin Island
	*Desmarestia anceps*	Ipanema
	*Desmarestia antarctica* R.L. Moe and P.C. Silva	Ipanema
Rhodophyta		
Gracilariaceae	*Curdiea racovitzae* Hariot	EACF
	*Curdiea racovitzae*	Punta Plaza
Callithamniaceae	*Georgiella confluens* (Reinsch) Kylin	Penguin Island
Gigartinaceae	*Sarcopeltis antarctica* Hommersand, Hughey, Leister and P.W. Gabrielson	EACF
	*Sarcopeltis antarctica*	Penguin Island
	*Sarcopeltis antarctica*	Punta Plaza
	*Iridaea cordata* (Turner) Bory de Saint-Vincent	EACF
	*Iridaea cordata*	Penguin Island
	*Iridaea cordata*	Ipanema
	*Iridaea cordata*	Punta Plaza
Delesseriaceae	*Myriogramme mangini* (Gain) Skottsberg	Punta Plaza
Palmariaceae	*Palmaria decipiens* (Reinsch) R.W.Ricker	Penguin Island
	*Palmaria decipiens*	Ipanema
	*Palmaria decipiens*	Yellow Point
Plocamiaceae	*Plocamium cartilagineum* (Linnaeus) P.S.Dixon	EACF
	*Plocamium cartilagineum*	Penguin Island
	*Plocamium cartilagineum*	Punta Plaza
Bangiaceae	*Pyropia endiviifolia* (A. Gepp and E. Gepp) H.G. Choi and M.S. Hwang	Penguin Island
	*Pyropia endiviifolia*	Punta Plaza

EACF (“Estação Antártica Comandante Ferraz”)

### 2.2 Seaweed Extracts Preparation

The seaweed samples were subjected to an identical extraction process. The frozen samples were freeze-dried, triturated, and extracted three times with ethyl acetate and methanol (EtOAc:MeOH 1:1 v/v) (HPLC, Tedia) at a proportion of 3.5 mL of solvent mixture per 1 g of sample (dry weight). The samples were left at room temperature for 72 h and assisted by ultrasound (LogenScientific, LS-218) for 20 min each time. After extraction, the samples were filtered, and the solvents were removed by evaporation under reduced pressure at 40 °C to obtain dry extracts.

### 2.3 Antimycobacterial Activity

#### 2.3.1 Growth of Microorganisms

Antimycobacterial activity of seaweed extracts was determined against two strains of Mtb that differed in virulence levels: a strain with low virulence (H37Rv) and a highly virulent strain (Mtb M299; isolated from a patient with TB in Mozambique). Strains were grown in Middlebrook 7H9 broth containing 10% albumin-dextrose-catalase complex (ADC, Difco Laboratories) and 0.05% Tween 80 in the presence of 5% CO_2_ at 37 °C for 7 days until log phase under biosecurity level 3 conditions.

#### 2.3.2 Minimum Inhibitory Concentration

Minimum inhibitory concentrations (MICs) of the samples were determined using the 3-(4,5-dimethylthiazol-2-yl)-2,5-diphenyltetrazolium bromide (MTT) assay.^20^ This method evaluates the functionality of viable mycobacteria using a tetrazolium salt, which is converted to formazan crystals after being metabolized by dehydrogenase enzymes.

Bacterial suspensions (1 × 10^6^ CFU) were then added to each well containing samples at concentrations of 100, 20, 4, and 0.8 μg/mL and to each of the growth-control wells. The sealed plates were incubated for 5 days at 37 °C and 5% CO_2_ for both strains.^21^ MTT solution (5 mg/mL) was added to each well, and the plates were incubated for 3 h. The cells were then treated with lysis buffer (20% w/v sodium dodecyl sulfate (SDS)/50% dimethylformamide (DMF) in distilled water, pH 4.7). Measurements were performed using a plate spectrophotometer (Dynatech MR5000) at 570 nm. As a positive control, the bacterial suspension was treated with rifampicin at concentrations ranging from 0.032 to 10 μg/mL. A bacterial suspension without any treatment was used as a negative control.

### 2.4 Cytotoxicity Testing in Macrophages

#### 2.4.1 Cell Culture

Murine macrophage cell line RAW 264.7 (American Type Culture Collection), RRID:CVCL_0493 was grown in Dulbecco’s modified Eagle’s medium (DMEM-F12, Gibco) supplemented with 10% Fetal Bovine Serum (FBS) and gentamicin (1 μg/mL) in the presence of 5% CO_2_ at 37 °C.

For the experiments, cells were plated in 96-well plates (5 × 10^4^ cells/well) and incubated for 24 h in the presence of 5% CO_2_ at 37 °C to ensure macrophage adhesion and culture stability. For macrophage stimulation, cell cultures were treated with lipopolysaccharide (LPS, 1 μg/mL) (*Escherichia coli* O55:B5; Sigma-Aldrich, USA) and incubated with seaweed samples at concentrations of 100, 20, 4, and 0.8 μg/mL for another 24 h. A portion of the supernatant was collected to determine its ability to inhibit the inflammatory mediator Nitric Oxide (NO).

#### 2.4.2 MTT Assay

The cytotoxic effects of seaweeds on cell viability in RAW 264.7 cultures stimulated with LPS were determined by using the MTT assay as previously described. After incubation, the supernatant was discarded and formazan crystals were dissolved in isopropanol with the addition of hydrochloric acid (HCl) (4 mM). Measurements were performed at 570 nm by using a plate spectrophotometer (Dinatech MR500). Macrophages stimulated with LPS served as negative controls for cell death, whereas macrophages stimulated with LPS and treated with Triton X-100 (1%) were used as positive controls. The effect of dimethyl sulfoxide (DMSO, Sigma-Aldrich), the solvent used in the suspension of the samples, was also evaluated to ensure that the solvent did not affect cellular cytotoxicity.

Cell death was expressed as percent inhibition and calculated using the following formula: percent inhibition = 100 – (100­(sample – negative control)/(positive control – negative control)). IC_50_ values were calculated by nonlinear regression analysis of concentration/inhibition curves using GraphPad Prism 6 with a sigmoidal slope dose–response variable. Results are expressed as means with the corresponding 95% confidence intervals (CIs) from three independent experiments performed in triplicate.

### 2.5 Inhibition of Nitric Oxide Production

The inhibition of the production of the inflammatory mediator NO was assessed indirectly by measuring the concentration of nitrite in the supernatant. The previously collected supernatant was transferred to a new 96-well plate to which Griess reagent (1% *p*-aminobenzenesulfonamide + 0.1% naphthylethylenediamine dihydrochloride in 5% phosphoric acid, Sigma-Aldrich) was added.^22^ After 10 min, the absorbance was measured using a plate spectrophotometer at 540 nm. As a reference to produce NO, a standard curve was prepared with sodium nitrite diluted to concentrations from 0.78 to 100 μM. The standard drug L-NMMA, the acetate of NG-methyl-l-arginine (Sigma-Aldrich), was used as a positive control.

### 2.6 Chemical Profile of the Crude Extract Using Gas Chromatography–Mass Spectrometry (GC-MS) and a Molecular Networking Approach

Prior to GC-MS injection, each crude extract was diluted in dichloromethane (HPLC, Tedia) and filtered using a 0.45-μm PTFE syringe filter (Millipore, USA) to remove any insoluble constituents. The solvent was evaporated, and the samples were lyophilized. The remaining material was resuspended in ethyl acetate (HPLC grade, Tedia) to a final concentration of 1 mg/mL. Analysis by GC-MS was carried out on a GC-MS QP2010-Ultra (Shimadzu), with an AOC-20i autosampler and a capillary column RTx-1MS (30 m × 0.25 mm; film thickness: 0.1 μm; Restek Corp, Bellefonte, PA, USA). A sample (1 μL) was injected at 280 °C and a split ratio of 5. Helium (He) was used as the carrier gas at a flow rate of 1 mL/min with the following temperature ramp: The initial temperature of 120 °C was maintained for 2 min, with a ramp of 6 °C per minute to 305 °C, which was maintained for 3 min, giving a total analysis time of 36 min. The ionization source was maintained at 200 °C and the interface at 305 °C. Peak identification of the crude extracts was performed by comparing the relative retention index (RI) with library margins (NIST 08 and Wiley 7).

Data from GC-MS were processed before the spectral library search, and molecular networking workflow was performed. The files of the analysis GC-MS were saved in the file format CDF. The data were processed using the MSHub deconvolution workflow^23^ to generate a spectral file (.mgf) and a quantification table (.csv), which were directly used as the input for the Molecular Library Search GC workflow. A molecular network was then constructed using GNPS (ver. 30), filtering edges with a cosine score greater than 0.6 and at least six matched peaks. Furthermore, edges between two nodes were retained in the network only if each of the nodes appeared in the respective top 10 most similar nodes of the other. Library spectra were filtered in the same way as the input data, and all matches between network spectra and library spectra had to have a score above 0.6 and at least six matching peaks. The generated molecular network files (.graphml) were visualized and analyzed in Cytoscape (version 3.8),^24^ and the nodes corresponding to the control were removed manually.

## 3 Results and Discussion

### 3.1 Antimycobacterial, Immunomodulatory, and Cytotoxic Activities

Despite the remarkable biomass and biodiversity of Antarctic seaweed, relatively few studies have explored the pharmacological potential of their secondary metabolites.^15^ In the present study, 29 extracts obtained from 15 Antarctic seaweed species belonging to the genera Chlorophyta (two species, 6.9%), Ochrophyta (eight species, 27.6%), and Rhodophyta (19 species, 65.5%) were evaluated *in vitro* for their antimycobacterial and NO-inhibitory potential. These species were collected from different locations on the Antarctic Peninsula. In addition, cytotoxicity was assessed in RAW 264.7 murine macrophages to determine the extract selectivity. The MIC_50_, MIC_90_, IC_50_, and CC_50_ values are summarized in Table 2.

**Table 2:** Antimycobacterial, Immunomodulatory, and Cytotoxic Activities of Antarctic Seaweed Used in This Study

		Mtb H37Rv		Mtb M299		NO	Cytotoxicity
Species	Collection Sites	MIC_50_ (μg/mL)	MIC_90_ (μg/mL)	MIC_50_ (μg/mL)	MIC_90_ (μg/mL)	IC_50_ (μg/mL)	CC_50_ (μg/mL)
Chlorophyta							
*Monostroma hariotii*	Penguin Island	4.7 ± 1.2	73.6 ± 1.2	46.9 ± 0.7	>100	10.5 ± 1.1	38.3 ± 0.8
*Spongomorpha arcta*	Penguin Island	21.4 ± 1.6	>100	60.6 ± 0.8	>100	12.9 ± 1.3	35.2 ± 1.3
Ochrophyta							
*Adenocystis utricularis*	Penguin Island^a^	34.4 ± 1.9	>100	>100	>100	23.2 ± 1.4	90.9 ± 1.2
	Penguin Island^b^	11.8 ±1.2	>100	>100	>100	17.7 ± 1.2	34.9 ±1.1
	Punta Plaza	>100	>100	>100	>100	6.3 ± 1.2	85.9 ± 1.2
*Ascoseira mirabilis*	Penguin Island	17.8 ± 0.5	>100	74.3 ± 0.9	>100	26 ± 1.4	27.2 ± 1.4
*Desmarestia anceps*	EACF	74.9 ± 1.8	>100	97.8 ± 1.4	>100	5.6 ± 0.9	41.5 ± 1.1
	Penguin Island	4.8 ± 2.0	>100	6.1 ± 2.2	>100	3.7 ± 1.2	9.7 ± 1.2
	Ipanema	6.4 ± 1.1	15.3 ±1.2	74.5 ±1.2	>100	6.4 ± 1.2	37.3 ± 1.1
*Desmarestia antarctica*	Ipanema	2.8 ± 0.3	98.5 ± 1.5	10.5 ± 0.6	>100	19.6 ± 0.8	42.4 ± 1.4
Rhodophyta							
*Curdiea racovitzae*	EACF	17.4 ± 1.1	>100	48.8 ± 1.7	>100	13.0 ± 1.3	>100
	Punta Plaza	2.9 ± 0.3	42.6 ± 1.6	10.6 ± 0.6	>100	8.2 ± 1.2	70.1 ± 1.0
*Georgiella confluens*	Penguin Island	5.8 ± 0.8	40.2 ± 1.3	16.8 ± 0.9	>100	19.5 ± 1.3	42.4 ± 1.4
*Sarcopeltis antarctica*	EACF	8.8 ± 0.9	75.5 ± 1.2	11.8 ± 0.9	>100	7.2 ± 1.2	42.3 ± 0.9
	Penguin Island	2.6 ± 0.4	>100	6.6 ± 1.3	>100	10.1 ± 1.3	>100
	Punta Plaza	30.7 ± 0.9	>100	46.1 ± 2.0	>100	33.8 ± 1.0	>100
*Iridaea cordata*	EACF	10.4 ± 1.5	>100	33.6 ± 1.5	>100	4.7 ± 1.2	45.6 ± 1.4
	Penguin Island	9.9 ± 0.2	64.7 ± 1.2	53.7 ± 0.4	>100	20.3 ± 1.4	>100
	Punta Plaza	2.8 ± 0.2	>100	74.6 ± 1.5	>100	16.4 ± 1.1	37.3 ± 1.2
	Ipanema	22.5 ± 1.2	>100	74.9 ± 1.3	>100	12.3 ± 0.7	61.8 ± 1.0
*Myriogramme mangini*	Punta Plaza	73.5 ± 1.2	>100	78.9 ± 1.3	>100	10.1 ± 1.3	>100
*Palmaria decipiens*	Penguin Island	41.1 ± 1.0	>100	88.4 ± 1.1	>100	6.2 ± 1.2	34.5 ± 1.4
	Ipanema	23.9 ± 1.5	>100	77.4 ± 0.7	>100	19.2 ± 0.3	>100
	Yellow Point	>100	>100	>100	>100	10.1 ± 1.2	33.2 ± 0.9
*Plocamium cartilagineum*	EACF	11.2 ± 1.5	>100	13.1 ± 1.4	>100	6.3 ± 1.1	39.8 ± 0.7
	Penguin Island	93.1 ± 2.1	>100	>100	>100	15 ± 1.0	42.6 ± 0.9
	Punta Plaza	13.6 ± 1.1	>100	35.0 ± 1.8	>100	7.3 ± 1.3	47.8 ± 1.1
*Pyropia endiviifolia*	Penguin Island	4.6 ± 0.8	78.7 ± 1.5	17.6 ± 0.8	>100	8.8 ± 1.0	42.6 ± 1.3
	Punta Plaza	2.1 ± 0.2	15.3 ± 1.3	3.2 ± 0.7	50.6 ± 1.3	6.8 ± 1.3	24.0 ± 0.9
Rifampicin		0.2 ± 0.1		1.1 ± 0.1			

a Young stage;

b Adult stage. Data are the mean ± standard deviation (*n* = 3).

According to the Clinical and Laboratory Standards Institute (CLSI), crude extracts with MIC_50_ values below 128 μg/mL and pure compounds with MIC_50_ values below 25 μM are considered promising for antimycobacterial drug discovery.^25,26^ Based on these criteria , all samples, except *Adenocystis utricularis* from Punta Plaza and *Palmaria decipiens* from Yellow Point, exhibited relevant antimycobacterial activity against the reference strain Mtb H37Rv. Notably, twenty-one extracts (72.4%) displayed MIC_50_ values below 25 μg/mL, and nine (31%) showed MIC_90_ values below 100 μg/mL. Furthermore, nine extracts (31%) also showed significant activity against the hypervirulent clinical isolate M299, with MIC_50_ values below 25 μg/mL.

Among all extracts, the *Pyropia endiviifolia* collected from Punta Plaza, exhibited the most potent activity against both strains, with MIC_50_ values of 2.1 ± 0.2 and 3.2 ± 0.7 μg/mL against H37Rv and M299, respectively. Additionally, extracts of *Desmarestia antarctica*, *D. anceps*, *Curdiea racovitzae*, *Sarcopeltis antarctica*, *Gigartina skottsbergii*, and *Iridaea cordata* showed notable activity against both Mtb strains, highlighting their potential as sources of novel anti-TB agents.

In addition to their antimycobacterial activity, extracts from Antarctic seaweeds showed the ability to modulate the production of NO. The formation of the mediator NO by inflammatory macrophages due to the stimulation of inducible NO synthase (iNOS) is one of the mechanisms of host defense against intracellular pathogens, such as Mtb.^27^ Although NO production plays an important role in the initial phase of infection, enabling macrophages to kill phagocytosed bacilli, excessive and continuous production can lead to NO-mediated tissue damage in the late or chronic stages of TB.^28^ In these specific cases, mainly related to MDR and XDR strains, control of immunopathology using host-directed therapy is desirable and recommended.^29^

In this context, agents with antimycobacterial and anti-inflammatory properties may represent important alternatives for TB-associated pulmonary hyperinflammation. Seaweeds are a source of several known bioactive compounds, but few studies have described the anti-inflammatory activity or immunomodulatory properties of Antarctic organisms, including seaweeds. Therefore, in the present study several extracts exhibited effective inhibition of NO production in lipopolysaccharide (LPS)-stimulated RAW 264.7 cells, suggesting anti-inflammatory potential. A concentration–response curve was generated for each extract, and the IC_50_ values are shown in Table 2.

All Antarctic seaweed extracts, except for *A. mirabilis* from Penguin Island and *S. antarctica* from Punta Plaza, showed a significant inhibitory effect on the production of NO with IC_50_ values below 25 μg/mL. Among the promising samples in terms of NO inhibition are *D. anceps*, *C. racovitzae*, *S. antarctica*, *I. cordata*, *P. cartilagineum*, and *P. endiviifolia*, with IC_50_ values ranging from 3.7 ± 1.2 to 8.8 ± 1.0 μg/mL, suggesting dual activity for these samples, which can be advantageous for the treatment of severe cases of TB.

Considering that some drugs with antimycobacterial activity may be toxic to normal cells and damage them over time,^30^ it is important to evaluate the cytotoxic activity of the extracts on normal cells. In addition to their antimycobacterial and anti-inflammatory activities, the toxicity of the extracts was evaluated in RAW 264.7 macrophages, which are Mtb host cells. Most of the extracts exhibited low toxicity in these cells, suggesting a promising safety profile for their potential therapeutic applications. As shown in Table 2, the seaweed extracts exhibited a variety of cytotoxic effects, with CC_50_ values ranging from 9.7 ± 1.2 to >100 μg/mL.

According to the CLSI criteria, compounds with SI values ≥10 are considered promising for further anti-TB drug development.^31,32^ In this context, it was observed that some evaluated extracts exhibited promising antimycobacterial activity with low cytotoxicity. *S. antarctica* demonstrated an excellent safety profile, showing a favorable selectivity index (SI) >10 against both tested strains of Mtb, H37Rv and M299. Additionally, five other extracts (*D. antarctica* from Ipanema, *I. cordata* from Penguin Island, and *C. racovitzae*, *I. cordata*, and *P. endiviifolia* from Punta Plaza) also displayed SI values >10, suggesting promising potential.

Our findings revealed marked geographic variation in the antimycobacterial activity of *Plocamium cartilagineum*. Extracts collected from EACF and Punta Plaza exhibited potent activity against Mtb H37Rv, with MIC_50_ values of 11.2 and 13.6 μg/mL, respectively. The EACF extract also showed strong activity against the hypervirulent M299 strain (MIC_50_ = 13.1 μg/mL), whereas the Punta Plaza extract displayed moderate activity (MIC_50_ = 35.0 μg/mL). In contrast, the Penguin Island sample was substantially less active against both strains. These findings highlight pronounced intraspecific chemical variability associated with the collection site, reinforcing the influence of geographic and environmental factors on the production of bioactive metabolites in Antarctic seaweeds.

Our results corroborate the study by König et al. (2000), wherein a polyhalogenated cyclic monoterpene from the red alga *P. cartilagineum* collected from Deception Island, Antarctic Peninsula, exhibited activity against Mtb H37Rv and *M. avium* at concentrations of 32 and 34 μg/mL, respectively.^33^ Furthermore, *D. anceps*, whose anti-inflammatory properties were previously assessed by Moles et al. (2014) demonstrated inhibition of the inflammatory mediator prostaglandin (PGE2) at noncytotoxic concentrations (50 and 125 μg/mL).^34^ These results are supported by previous studies that have identified bioactive compounds in seaweed with anti-inflammatory properties, suggesting the therapeutic potential of these extracts in the treatment of chronic inflammatory diseases such as TB.^29,35–38^ However, most of these studies have concentrated on a limited number of genera from tropical or subtropical seaweed species. The results obtained from our study on the antimycobacterial activity of Antarctic seaweeds are complemented and contextualized by previous research, providing valuable insights into the potential pharmacological applications of these organisms, such as those conducted by Berneira et al. (2021) and Martín-Martín et al. (2022) who demonstrated the inhibitory effects of extracts from *Curdiea racovitzae*, *Adenocystis utricularis*, *D. anceps*, and *D. menziesii* against pathogenic bacteria including *Escherichia coli*, *Staphylococcus aureus*, *Bacillus cereus*, and *Salmonella typhimurium*, indicating their broad-spectrum antimicrobial properties.^16,39^ Moreover, Martins et al. (2018) highlighted the antifungal activity of an extract from *Himantothallus grandifolius* against fluconazole-resistant fungal strains.^40^ Similarly, Martorell et al. (2021) reported significant fungal inhibition by extracts from *P. cartilagineum*, *Georgiella confluens*, *D. anceps*, *D. Antarctica*, *D. menziesii*, and *H. grandifolius* showing promising results against various fungal genera.^41^ Furthermore, bioactivity-guided fractionation studies on *D. antarctica* and *I. cordata* by dos Santos et al. (2020) and Rangel et al. (2019) revealed potent antiparasitic effects against *Leishmania amazonensis*, suggesting their potential as novel therapeutic agents against parasitic infections.^11,42^ Maschek et al. (2011) conducted a bioassay-guided fractionation of extracts from *S. antarctica*, revealing significant inhibition of influenza replication, underscoring the antiviral potential of Antarctic seaweeds.^43^ Additionally, Ponce et al. (2003) and Pujol et al. (2006) demonstrated the antiviral activity of compounds derived from *A. utricularis* and *S. antarctica* against herpes simplex viruses, further highlighting the pharmacological relevance of Antarctic seaweeds in combating viral infections.^44,45^ Collectively, these findings highlight the remarkable pharmacological potential of Antarctic seaweeds and reinforce their value as promising sources of novel bioactive compounds for the treatment of tuberculosis and other infectious diseases.

### 3.2 Chemical Composition Analysis of the Bioactive Extracts

Chromatograms obtained by GC-MS analysis (retention time, 3.5 to 35 min) of lipophilic extracts from six promising Antarctic seaweeds—including *Desmarestia antarctica* from Ipanema; *C. racovitzae*, *I. cordata*, and *P. endiviifolia* from Punta Plaza; and *S. antarctica* and *I. cordata* from Penguin Island—are depicted in Figure 1.

**Figure 1:**
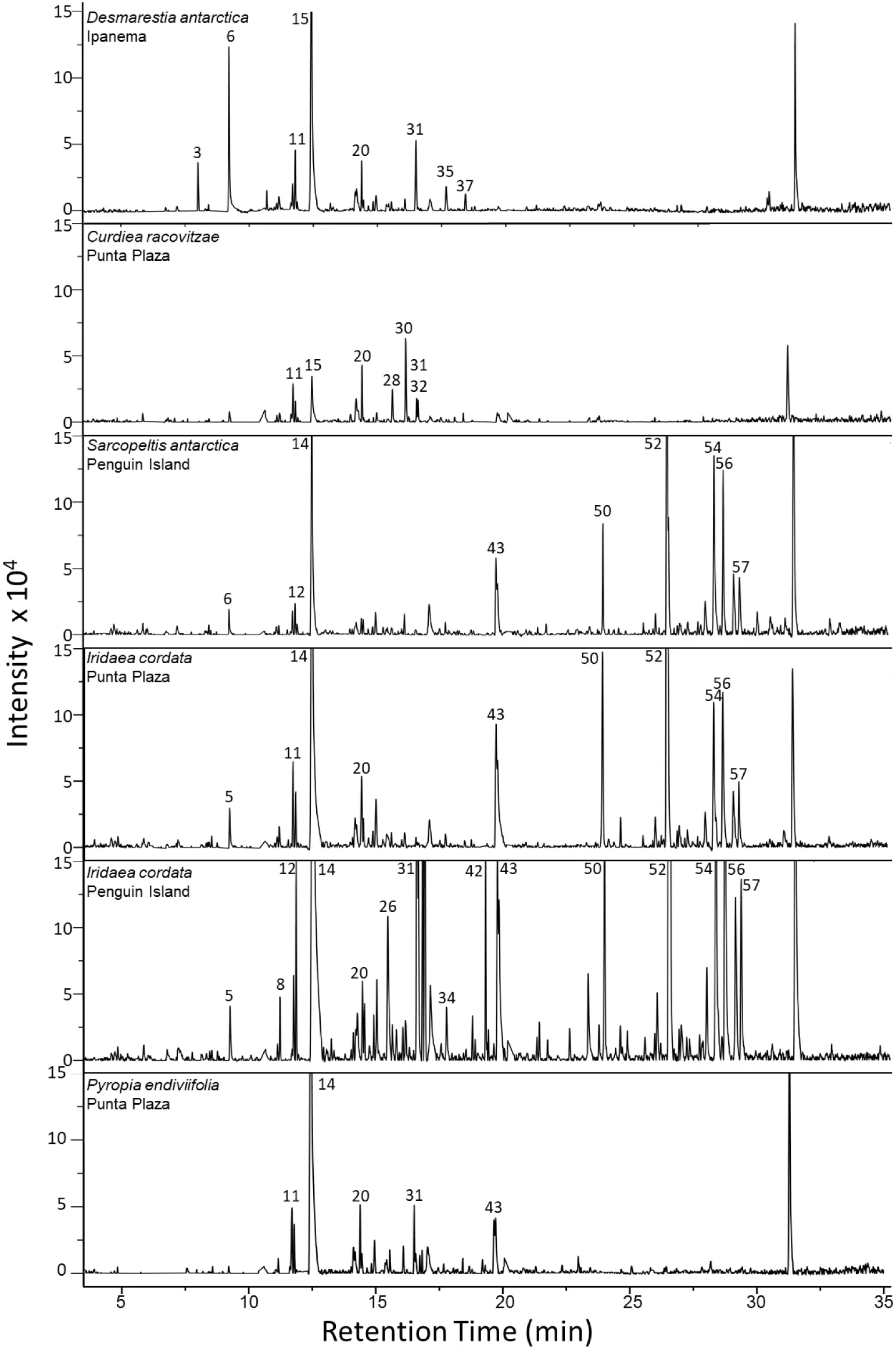
Chromatograms obtained by GC-MS analysis (t_R_, 3.5 to 35 min) of six promising seaweeds samples for tuberculosis, including *Desmarestia antarctica* from Ipanema, *Curdiea racovitzae* and *Iridaea cordata* from Punta Plaza, and *Sarcopeltis antarctica*, *Iridaea cordata*, and *Pyropia endiviifolia* from Penguin Island. Numbers indicate significant peaks corresponding to identified compounds described in the text and Table 3.

To characterize the compounds responsible for the observed antimycobacterial activity, GC-MS data from all twenty-nine samples collected across five locations on the Antarctic Peninsula were submitted to the GNPS platform for molecular network construction. Molecular Network (MN) analysis revealed a total of 187 nodes, including 58 singleton nodes that remained unannotated in the GNPS spectral library. Nodes detected in the blank sample (*n* = 5) were excluded from further analysis. The resulting network comprising 124 interconnected nodes, each associated with qualitative, quantitative, and metadata information, was organized into 12 molecular families (F1–F12) formed by at least two ions with similarity in the MS, using a cosine similarity threshold of 0.60 (Figure 2).

**Figure 2:**
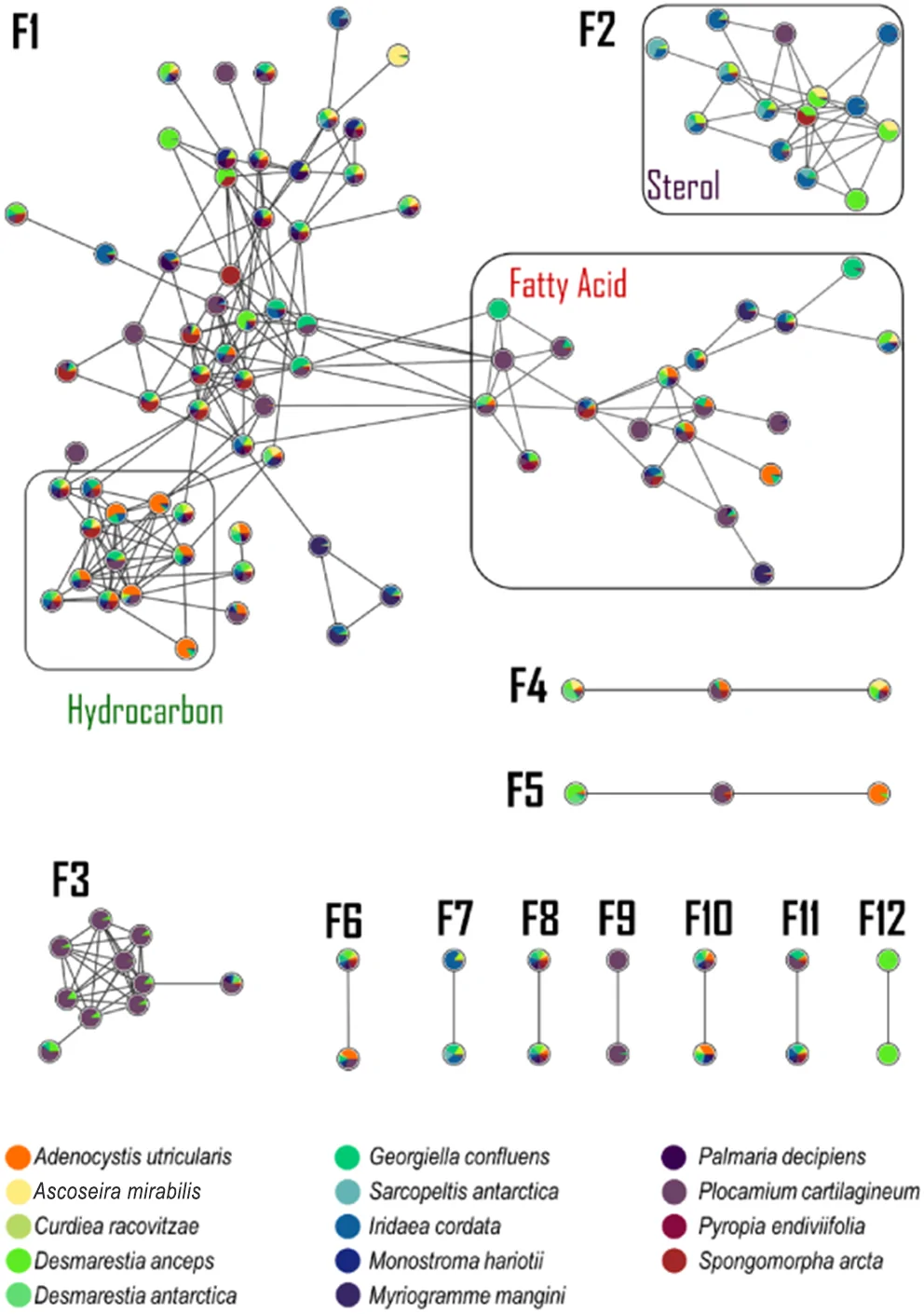
Molecular network generated from GC-MS data of Antarctic seaweed extracts collected on the Antarctic Peninsula, processed via the GNPS platform, and visualized using Cytoscape. Only clusters containing two or more nodes are shown. Nodes corresponding to background signals (mobile phase blanks) were excluded. Three major annotated molecular families—hydrocarbons, fatty acids, and sterols—are highlighted. Node colors represent different seaweed samples.

Among these, F1 contained the largest number of nodes (76 nodes), followed by F2 (14 nodes) and F3 (10 nodes), whereas F4 to F12 comprised only two or three nodes each. Based on GNPS library matching and manual inspection using the NIST 08 and Wiley 7 mass spectral databases, 60 compounds were annotated in the bioactive extracts (Table 3). Fatty acids, fatty acid-derived compounds, hydrocarbons, and sterols were the predominant chemical classes identified in promising samples.

**Table 3:** Principal Putative Constituents Annotated by the GC-MS Database of Promising Anti-TB Seaweed Samples^c^

			Peak area (%)					
Compound No.	t_R_ (min)	Putative Annotated Compound	*Desmarestia antarctica* Ipanema	*Curdiea racovitzae* Punta Plaza	*Sarcopeltis antarctica* Penguin Island	*Iridaea cordata* Punta Plaza	*Iridaea cordata* Penguin Island	*Pyropia endiviifolia* Punta Plaza
1	4.83	2,2,4,4,6,8,8-Heptamethylnonane^a^			0.23	0.19	0.08	0.17
2	7.74	Methyl vinyl ketone^b^					0.05	0.10
3	8.07	Loliolide^a^	3.90					
4	8.42	Pentadecane^a^			0.23	0.09	0.06	0.22
5	9.24	Dodecanoic acid^a^	1.27	0.37	0.31	0.61	0.4	0.18
6	9.24	6,7-Epoxyoctadecanoic acid methyl ester^a^	12.35	1.31	0.52	0.20	0.03	0.14
7	11.09	2-Hexenal^b^		0.72	0.28	0.29	0.12	
8	11.18	Diisobutyl phthalate^a^	1.04	1.16	0.17	0.29	0.28	0.58
9	11.18	2,6-Ditert-butyl-4-methylphenol^a^					0.08	
10	11.67	cis-1-Chloro-9-octadecene^b^	1.35	1.08		0.07		0.30
11	11.72	9-Eicosene^b^	3.38	4.61	0.60	1.48	0.55	2.48
12	11.82	Octadecanoic acid, methyl ester^a^	3.20	2.50	0.80	0.93	1.28	1.73
13	11.91	1,2-Benzenedicarboxylic acid hexyl octyl ester^b^			0.27			
14	12.45	Hexadecanoic acid^a^	2.36	3.42	14.53	18.42	20.32	44.85
15	12.45	Pentadecanoic acid^a^	31.76	10.30	4.58	3.05	0.64	3.98
16	13.02	1-Pentadecene^a^					0.11	
17	14.15	Tridec-4-en-2-ynal^b^	1.64	1.27		0.74	0.05	1.15
18	14.21	N-pentadecanol^a^	1.13	1.50	0.64	0.31	0.20	0.38
19	14.21	Dioctadecyl phospite^a^	1.15			0.05		0.16
20	14.41	1-Nonene^a^	3.50	9.65	0.47	1.26	0.47	3.03
21	14.48	3-Chloropropionic acid, hexadecyl ester^b^	0.66			0.55	0.35	0.85
22	14.67	2-Octyl-1-dodecanol^b^	0.24	0.49	0.14	0.12		
23	14.86	Decanoic acid, methyl ester^a^	0.52	0.52	0.19	0.21	0.28	0.39
24	15.27	1,3-Propanediol, ethyl tetradecyl ether^a^	0.71			0.05		
25	15.40	Hexanedioic acid, dioctyl ester^a^	0.59			0.36	0.13	
26	15.40	Octadecanoic acid^a^			0.36	0.15	1.71	0.44
27	15.57	3-Methyl-2,6-dioxo-4-hexanoic acid^b^	1.21	1.52	0.12	0.17		1.2
28	15.57	1-Octene^a^		2.09	0.32			0.16
29	15.96	9-Octadecenoid acid^b^					0.25	
30	16.09	Heptadecane^a^		18.34	0.73	0.51	0.61	1.59
31	16.52	Acetylcitric acid, tributyl ester^a^	4.06	3.23		0.14	7.29	2.28
32	16.58	4-Hidroxytetradec-2-ynal^b^		1.70		0.22	1.32	1.17
33	16.75	Eicosanoid acid, 2-(acetyloxy)-1-((acetiloxy)methyl))ethyl ester^b^				0.08	2.77	0.57
34	16.84	Hexadecanoid acid, 2,3-bis(acetyloxy) propyl ester^b^					4.34	0.82
35	17.08	Thiosulfuric acid, S-(2-aminoethyl)ester^b^	1.27		2.06	0.89	0.78	1.53
36	17.70	Butane, 1,1-bis(ethenyloxy)^b^			0.33			
37	17.70	Oxalic acid, allyl pentadecyl ester^b^	1.84			0.20	0.35	0.25
38	18.44	Hexanedioic acid, bis(2-ethylhexyl) ester^a^	0.99	1.15		0.11	0.05	0.54
39	18.70	9-Hexacosene^b^				0.13	0.18	
40	18.70	9-Tricosene^b^				0.22	0.08	
41	18.81	Sulfurous acid, octadecyl-2-propyl ester^b^					0.06	
42	19.22	Dodecanoic acid, 3-methylbutyl ester^a^					1.57	0.65
43	19.70	Hexadecanoic acid, 2-hidroxy-1-(hidroxymethyl) ethyl ester^a^	0.32	1.30	4.30	5.21	1.94	2.48
44	19.70	Docosyl octyl ether^a^	0.14			0.41	2.24	0.33
45	21.19	Cis-(2)-nonadecene^b^					0.15	
46	22.50	1-Pentene^a^					0.11	
47	22.50	2-Methyl-1-pentene^a^					0.11	
48	23.65	3-Isopropyl-6,10-dimethylundecane-2-ol^a^	1.06				0.11	
49	23.65	3,7,11,15-tetramethyl-1-hexadecen-3-ol^b^					0.17	
50	23.86	4,6-Cholestadien-3beta-ol^b^			3.52	3.04	2.44	
51	25.82	1-Nonene^a^				0.88	0.26	
52	26.37	Cholesterol^a^			17.68	28.32	13.63	0.22
53	26.37	Cholesteryl benzoate^a^			2.90	1.59	0.27	
54	28.21	Stigmasterol^a^			8.16	4.35	5.39	0.55
55	28.43	Isofucosterol^b^					0.25	
56	28.57	Stigmast-5-ene-3beta-ol^a^			7.88	5.07	5.31	
57	29.21	Cholest-5-en-7-one,3 hidroxy^b^			2.67	2.25	1.94	
		Area identified	81.64	68.23	74.99	83.21	81.16	75.47

a Hybrid approach;

b NIST08 or Wiley 7 libraries.

c t_R_, retention time.

Cluster F1, the largest molecular family, was dominated by fatty acids and hydrocarbons, which are compound classes frequently reported in Antarctic seaweeds.^9,16,42,46,47^ Within this cluster, the fatty acid family comprised 20 nodes, including four fatty acids (**5**, **14**, **15**, and **26**) and three fatty acid esters (**25**, **38**, and **42**) identified in promising samples. Notably, hexadecanoic acid (**14**) and pentadecanoic acid (**15**) were among the major constituents. The identified esters included hexanedioic acid dioctyl ester (**25**) and dodecanoic acid, 3-methylbutyl ester (**42**).

Fatty acids play a central role in mycobacterial physiology, serving as essential precursors for the biosynthesis of mycolic acids and other complex lipids that constitute the highly hydrophobic cell envelope of Mtb.^48^ Several saturated fatty acids have demonstrated significant antimycobacterial activity, and a comprehensive overview of the biological activities reported for all major compounds identified in the promising extracts is presented in Supplementary Table S1. Hexadecanoic acid (palmitic acid—**14**), for example, exhibited MIC values of 100 and 50 μg/mL against the drug-susceptible H37Rv strain and multidrug-resistant clinical isolates of Mtb, respectively.^49^ In addition, hexadecanoic acid markedly enhanced the efficacy of rifampicin, reducing its MIC—ranging from 8- to 1000-fold—against multidrug-resistant *M. smegmatis* isolates, suggesting a promising adjuvant effect in antimycobacterial therapy.^50^ Several studies have demonstrated that fatty acids exhibit antimycobacterial effects either directly or through synergistic interactions with first-line drugs, supporting lipid metabolism as a vulnerable target in Mtb.^50,51^

Other saturated fatty acids have also shown potent activity against mycobacteria. Nonanoic, decanoic, and dodecanoic acids displayed MIC values as low as 50 μg/mL against Mtb H37Ra.^51^ Likewise, tetradecanoic acid and dodecanoic acid inhibited *M. smegmatis* by 90% and 76%, respectively, at 50 μg/mL,^52^ and their efficacy against *M. tuberculosis* H37Ra was confirmed in the study by Muniyan and Gurunathan (2016).^53^ Notably, fatty acids are believed to interfere with mycolic acid biosynthesis, disrupt cell wall integrity, and increase membrane permeability, thereby facilitating the intracellular penetration of antimycobacterial drugs.^54,55^ Unsaturated fatty acids, including oleic and linoleic acids, have likewise demonstrated activity against rapidly growing mycobacterial species such as *M. Aurum*, *M. phlei*, *M. smegmatis*, and *M. fortuitum*.^55^ Collectively, these findings support lipid metabolism and cell envelope biosynthesis as vulnerable targets in Mtb.

Because fatty acid uptake and assimilation are tightly regulated in Mtb, the disruption of lipid homeostasis can be detrimental to bacterial survival. Nazarova et al. (2018) demonstrated that controlled fatty acid transport is essential to prevent lipid toxicity during infection.^56^ In this context, the abundance of saturated fatty acids identified in the Antarctic seaweed extracts, particularly pentadecanoic and hexadecanoic acids, suggests that their antimycobacterial activity may result not only from their individual effects but also from a collective disruption of mycobacterial lipid metabolism and cell envelope homeostasis.

The identification of these fatty acids in Antarctic seaweed is consistent with previous chemical studies and reflects their ecological adaptation to cold environments.^57^ Moreover, their biological relevance extends beyond antimycobacterial activity, encompassing antibacterial, antitumoral, and broad-spectrum antimicrobial effects.^9,16,58^ For example, several fatty acids have demonstrated inhibitory action against methicillin-resistant *Staphylococcus aureus* (MRSA)^59^ and biofilm-forming fungi such as *Candida tropicalis*, including effects on biofilm structure and its associated virulence factors.^60^ Such activities further reinforce the therapeutic potential of these metabolites, particularly in the context of persistent infections and antimicrobial resistance.

The hydrocarbon molecular family comprised 13 nodes, of which seven compounds (**1**, **4**, **11**, **30**, **46**, **47,** and **51**) were annotated. These compounds were detected only in trace amounts in promising extracts, except for heptadecane (**30**), which was identified as a major constituent of *C. racovitzae*. Elevated levels of heptadecane have previously been reported in *C. racovitzae* collected from Punta Plaza (49.84%), where it was also described as a major metabolite, as well as in the Rhodophyta species *M. manguinii* (37.45%) and *G. confluens* (21.42%) from the Antarctic Peninsula, and in *Mazzaella laminarioides* (85.12%) from the sub-Antarctic region.^61,62^ Heptadecane has been associated with anti-inflammatory properties and potential therapeutic applications in NF-κB-mediated conditions in older adults,^63^ in addition to antimicrobial and antifungal activities.^64,65^ Its antibacterial potential has also been demonstrated in seaweed extracts against pathogenic bacteria.^66^ Furthermore, previous studies have identified a wide variety of hydrocarbons in Antarctic seaweeds—including aliphatic, branched, and cyclic alkanes, alkenes, and aromatic compounds—highlighting these organisms as a rich source of volatile compounds whose composition varies according to species and geographic origin.^67,68^

Molecular family F2 consisted exclusively of sterols and their derivatives, comprising 14 nodes. Among them, six compounds (**50**, **52**, **54**, **55**, **56,** and **57**) were annotated, with higher relative abundance in *I. cordata* and *S. antarctica*. The identified sterols included cholesterol (**52**), stigmasterol (**54**), and stigmast-5-ene-3β-ol (**56**), commonly known as β-sitosterol. These sterols are frequently reported in Antarctic seaweeds and are recognized for their diverse biological activities,^16,69,70^

In agreement with the present findings, de Santi et al. (2021), identified cholesterol as the major sterol in *I. cordata*, followed by β-sitosterol and stigmasterol.^69^ In *P. endiviifolia*, however, those authors also reported high levels of campesterol, brassicasterol, and fucosterol. In contrast, our analysis detected only cholesterol and stigmasterol in this species, both at low relative abundance. Although differences in extraction procedures and solvent polarity may partially explain these discrepancies, the observed variation likely reflects species-specific metabolic plasticity shaped by geographic origin, taxonomic identity, and ecological or physiological factors, including developmental stage,^19,71,72^

Cholesterol was identified as one of the major constituents of several of the most active extracts. Although cholesterol itself has not traditionally been regarded as a direct antimycobacterial agent, sterol-based compounds are increasingly being recognized as promising scaffolds against Mtb. Recent studies have demonstrated that oxidized cholesterol derivatives inhibit the growth of Mtb by targeting CYP125A1 and CYP142A1, two cytochrome P450 enzymes essential for cholesterol catabolism.^73^ Since cholesterol utilization is critical for mycobacterial survival, intracellular persistence, and virulence, disruption of this pathway can result in the accumulation of toxic metabolic intermediates and impaired bacterial growth.^56,74^ Accordingly, cholesterol may contribute, at least in part, to the antimycobacterial activity observed in the Antarctic seaweed extracts.

Sterols are widely recognized for their broad spectrum of biological activities, including antioxidant, antitumor, antibacterial, antiviral, antifungal, and anti-inflammatory effects.^70,75–77^ Among them, β-sitosterol and stigmasterol have demonstrated promising antimycobacterial activity. Previous studies reported MIC values of 25 μg/mL for β-sitosterol and 100 μg/mL for stigmasterol against Mtb H37Rv.^78^ Similar results were obtained by Saludes et al. (2002), who reported MIC values of 128 μg/mL and 32 μg/mL, respectively.^79^ Further underscoring the therapeutic potential of seaweed-derived sterols, saringosterol—a 1:1 mixture of the C-24 epimers 24R and 24S—originally isolated from the brown alga *Sargassum ringgoldianum* and later reisolated from *Lessonia nigrescens*, exhibited remarkable potency against Mtb H37Rv, with the 24R epimer displaying an MIC of 0.125 μg/mL.^80^

Another molecular family (F3), comprising 10 nodes, remained unannotated in the GNPS library at a cosine score threshold of 0.60. This cluster was primarily associated with *P. cartilagineum* and *D. anceps* specimens collected from Penguin Island. Given its taxonomic specificity and restricted geographic distribution, this molecular family represents a promising target for future bioactivity-guided fractionation and structural elucidation, aimed at identifying novel antitubercular metabolites.

Additional compounds representing diverse chemical classes were putative annotated through GNPS library matching and manual inspection of mass spectral databases:

Loliolide (compound **3**, F4), a monoterpene lactone and one of the major constituents of *Desmarestia antarctica*, has previously been reported in bioactive fractions of this species with antiparasitic potential.^42^ Beyond its antiparasitic potential, loliolide has demonstrated potent anti-inflammatory activity by suppressing NO production and inhibiting the release of proinflammatory cytokines, including TNF-α and IL-6, primarily through modulation of the Toll-like receptor-mediated NF-κB and MAPK signaling pathways.^38,81–85^ Altogether, these findings underscore the broad pharmacological potential of loliolide and its relevance as a bioactive metabolite from Antarctic seaweeds.
Diisobutyl phthalate (DIBP) (compound **8**, F5) was detected in all samples. Although this compound is often associated with anthropogenic contamination, its consistent occurrence across multiple biologically active Antarctic seaweed extracts suggests that endogenous biosynthesis may also contribute to its presence. Indeed, recent studies have proposed that certain phthalates, including DIBP, can be synthesized by marine organisms via pathways such as the shikimate route, particularly under environmental stress conditions.^62,86,87^ Phthalates exhibit a wide range of biological activities, including antimicrobial, cytotoxic, larvicidal, antiviral, antiparasitic, and apoptosis-inducing effects.^11,42,88,89^ Their detection in active extracts further supports their potential contributions to the observed biological activities.
Alkenes such as 9-eicosene (compound **11**, F1) and 1-nonene (compound **20**, F1) have previously been reported in seaweeds^90–93^ and are recognized for their antimicrobial properties.^94,95^

Octadecanoic acid (stearic acid) (compound **26**, F1) and its methyl ester, methyl stearate (compound **12**, F8), were identified in most of the promising extracts. Both compounds are commonly reported in seaweeds^96,97^ and have been associated with antimicrobial^98–101^ and anti-inflammatory activities.^102^ Fatty acid methyl esters (FAMEs), including methyl stearate, are known to inhibit microbial growth, further supporting their pharmacological relevance.^99^ Additionally, synergistic antiviral activity has been reported in combination with ribavirin.^103^
Acetylcitric acid tributyl ester (compound **31**, F1), also known as acetyl tributyl citrate (ATBC), is a citric acid ester with documented antimicrobial activity.^104,105^ It has also demonstrated antiparasitic potential,^106^ as well as antioxidant and anti-inflammatory effects,^107^ suggesting broad therapeutic applicability.
Thiosulfuric acid, S-(2-aminoethyl) ester (compound **35**, F1), was detected in all promising samples, except *C. racovitzae*. Previously reported in seaweeds,^108^ this compound has been associated with anti-inflammatory activity.^109^
Hexadecanoic acid, 2-hydroxy-1-(hydroxymethyl)­ethyl ester (compound **43**, F1), also known as 2-hexadecanoyl glycerol, palmitic acid β-monoglyceride, or palmitin-2-mono, was identified in all promising samples. This compound, previously reported in *D. antarctica*,^42^ has been associated with antifungal activity.^110,111^

The greatest chemical diversity was observed in the rhodophyte *I. cordata* collected from Penguin Island with 49 putative annotated compounds. In contrast, *C. racovitzae* *from* Punta Plaza exhibited the lowest diversity, with only 22 putative annotated compounds (Table 3). Across all bioactive extracts, fatty acids and sterols were the predominant chemical classes, a pattern commonly reported in polar seaweeds and closely associated with physiological adaptation to the extreme environmental conditions of Antarctica.

Fatty acids play a fundamental ecological role in Antarctic seaweeds, particularly by promoting membrane fluidity and ensuring cellular functionality under extreme polar conditions.^47^ Their abundance reflects adaptive biochemical strategies that optimize lipid composition in response to low temperatures and fluctuating environmental stressors.^47,112,113^ These adaptations also contribute to the regulation of intracellular reactive oxygen species (ROS), thereby minimizing oxidative stress and preserving photosynthetic efficiency.^114,115^

Beyond taxonomic affiliation, the chemical diversity and biological activity observed in this study were strongly influenced by both species identity and geographic origin. Pronounced intraspecific variation was evident in antimycobacterial activity, NO inhibition, and cytotoxicity among specimens collected from different sites. For example, *I. cordata* from Punta Plaza exhibited potent antimycobacterial activity (MIC_50_ = 2.8 μg/mL), albeit accompanied by relatively high cytotoxicity. Nevertheless, its selectivity index remained favorable (SI > 10), indicating selective activity against Mtb. The same species collected from Penguin Island also displayed considerable antimycobacterial potential, although slightly lower than the Punta Plaza specimen, but without detectable cytotoxicity, likewise yielding an SI above 10. In contrast, the Ipanema specimen showed only moderate activity (MIC_50_ = 22.5 μg/mL), coupled with moderate cytotoxicity and a low selectivity index (SI = 2.75), indicating limited therapeutic potential.

A similar geographic pattern was observed for *Sarcopeltis antarctica*, whose Penguin Island extract exhibited markedly greater antimycobacterial activity than those collected from EACF and Punta Plaza. Likewise, *C. racovitzae* demonstrated stronger activity in Punta Plaza than in EACF. Comparable site-dependent differences were also observed in *A. utricularis*, *D. anceps*, *P. decipiens*, and *P. cartilagineum*, further underscoring the strong influence of local environmental conditions on metabolite biosynthesis.

These findings are consistent with previous studies demonstrating that seaweed metabolite profiles are shaped by environmental variables, including nutrient availability, hydrodynamic conditions, temperature fluctuations, light intensity, and biotic interactions such as epiphytism and herbivory.^19,116–120^

In *S. antarctica* (formerly *Gigartina skottsbergii*), for example, pronounced biochemical differences have been documented between life stages. Gametophytes and tetrasporophytes produce distinct carrageenan types: the former predominantly synthesize κ- and λ-carrageenans, whereas the latter mainly produce λ-carrageenans.^121,122^ In addition, nonfertile fronds tend to accumulate higher levels of phycobiliproteins, whereas cystocarpic individuals contain greater amounts of proteins, floridean starch, and polysaccharides,^123^ highlighting the importance of physiological status in determining chemical profiles.

Environmental stressors, including temperature, salinity, and nutrient availability, further modulate metabolite production. In *S. antarctica*, increased nutrient availability, elevated temperatures, and reduced salinity levels are associated with greater accumulation of floridean starch and low-molecular-weight carbohydrates, likely representing a protective response to environmental stress.^123^ Similarly, Antarctic populations of *I. cordata* exhibit enhanced photosynthetic efficiency under low irradiance relative to Sub-Antarctic populations, indicating local adaptation to regional light and thermal regimes.^124,125^

More recently, Navarro et al. (2025) reported distinct physiological and biochemical responses to thermal stress between haploid and diploid stages of both *I. cordata* and *S. antarctica*, suggesting divergent ecological strategies.^126^ Such differences may influence not only primary metabolism but also secondary metabolite biosynthesis, ultimately affecting the biological activity.

Genetic variability also contributes substantially to the chemical diversity. Guillemin et al. (2018) documented considerable mitochondrial DNA variability in *I. cordata* populations across the Antarctic Peninsula. Although this variation did not reveal strongly differentiated genetic clusters, it suggests subtle intraspecific divergence that may underlie localized metabolic specialization.^127^ Likewise, Shilling et al. (2021) reported remarkable chemical polymorphism in *Plocamium spp*. within a radius of only 3.5 km at Palmer Station, driven by factors such as depth, irradiance, and hydrodynamics, all of which are closely linked to nutrient flux and biotic pressure.^128^

Ecological interactions and community structure provide an additional layer of complexity. Martín et al. (2016) observed that the abundance and biomass of *I. cordata* varied significantly among coastal sites at King George Island and could be reduced or excluded by the dominance of *A. utricularis*.^129^ Such competitive interactions can alter light availability, nutrient acquisition, and spatial distribution, thereby indirectly influencing metabolite biosynthesis. Similarly, Ko et al. (2021) showed that temporal and spatial fluctuations in Antarctic seaweed assemblages, including changes in *I. cordata* cover, reflect dynamic ecological pressures with important biochemical consequences.^130^

Collectively, these findings suggest that the antimycobacterial and anti-inflammatory activities observed among promising extracts are the result of a complex interplay of environmental conditions, life-stage differentiation, genetic variability, and ecological interactions. Integrating metabolomic, environmental, physiological, and genetic data sets will be essential to fully elucidate the regulatory mechanisms governing secondary metabolite production in Antarctic seaweeds and to maximize their biotechnological potential.

Despite growing ecological interest in Antarctic seaweeds, studies evaluating their antimicrobial potential remain limited. To our knowledge, this is the first study to investigate the antimycobacterial activity of Antarctic seaweeds. The observed bioactivities are likely associated with the presence of sterols, hydrocarbons, fatty acids, and fatty acid methyl esters identified by GC-MS, as well as with unidentified minor metabolites or potential synergistic interactions among these constituents.

In conclusion, Antarctic seaweeds represent a promising and still underexplored reservoir of bioactive compounds. Continued bioprospecting, coupled with comprehensive chemical and biological characterization of species from diverse Antarctic environments, may facilitate the discovery of novel therapeutic candidates for tuberculosis and other infectious diseases.

## 4 Conclusions

This study represents the first evaluation of the antimycobacterial potential of Antarctic seaweeds, revealing significant inhibitory activity against Mtb. Both chemical diversity and antimycobacterial efficacy were strongly influenced by taxonomic identity and geographic origin. Notably, 21 of 29 extracts exhibited MIC_50_ values below 25 μg/mL against the reference strain H37Rv, while nine extracts showed marked activity against the hypervirulent strain M299. Among these, extracts from *Pyropia endiviifolia* were particularly remarkable due to their exceptional potency.

Chemical profiles of the promising extracts revealed a predominance of fatty acids and sterols, suggesting that these metabolites, individually or through synergistic interactions, contribute substantially to the observed antimycobacterial activity. In addition to their antimicrobial effects, several extracts strongly inhibited NO production, indicating anti-inflammatory and immunomodulatory properties that may further enhance their therapeutic potential.

Given the limited knowledge regarding the biotechnological applications of Antarctic seaweeds, these findings provide an important foundation for future investigations. They further highlight the relevance of extreme marine environments as reservoirs of chemically unique and pharmacologically valuable natural products.

Overall, Antarctic seaweeds emerge as a promising and largely untapped source of novel antitubercular agents. Future studies should prioritize bioactivity-guided isolation, structural elucidation, and mechanistic investigations of the active constituents to advance their development as potential therapeutics for tuberculosis.

## Supplementary Material



## Data Availability

Data collected for this experiment are available upon request from the authors.

## Notes

Supplementary Table S1: Reported antimycobacterial activity of major compounds identified in promising samples by GC-MS (PDF)

## ○

Instituto de Investigación e Innovación en Energías Renovables y Medio Ambiente, Vicerrectorado de Investigación, Universidad Católica de Santa María de Arequipa, Arequipa 04013, Perú
