# Supplementary material for: Antimycobacterial Activity and Nitric Oxide Production Inhibition of Antarctic Seaweeds
Source: ACS Omega. 2026 Sep 2;11(36):53769–83. doi: 10.1021/acsomega.6c01332 (PMC13584166; doi:10.1021/acsomega.6c01332)
Supplement: Supplementary file 1 [file ao6c01332_si_001.pdf]

Antimycobacterial Activity and Nitric Oxide Production Inhibition of Antarctic Seaweeds

Carolina Rosas Fernández[1,2]*; Thatiana Lopes Biá Ventura Simão*[3]*; Elena B. Lasunskaia*[3]*; Ricardo Moreira Borges*[4]*; Pio Colepicolo*[5]*; Michelle F. Muzitano*[2]**; Angélica R. Soares*[1,6]**

[1]Grupo de Produtos Naturais de Organismos Aquáticos (GPNOA) – NUPEM, Universidade Federal do Rio de Janeiro– *Campus* Macaé, RJ, Brazil;

[2]Laboratório de Produtos Bioativos, Instituto de Ciências Farmacêuticas, Universidade Federal do Rio de Janeiro – *Campus* Macaé, RJ, Brazil;

[3]Laboratório de Biologia do Reconhecer, Centro de Biociências e Biotecnologia, Universidade Estadual do Norte Fluminense, Campos dos Goytacazes, RJ, Brazil;

[4]Instituto de Pesquisas de Produtos Naturais Walter Mors (IPPN), UFRJ, RJ, Brazil;

[5]Departamento de Bioquímica, Instituto de Química, Universidade de São Paulo, SP, Brazil;

[6]Laboratório de Produtos Naturais e Bioquímica, Instituto de Pesquisas do Jardim Botânico do Rio de Janeiro, RJ, Brazil.

Current Address for CRF: Instituto de Investigación e Innovación en Energías Renovables y Medio Ambiente, Vicerrectorado de Investigación, Universidad Católica de Santa María de Arequipa, Arequipa 04013, Perú

Supplementary Table S1. Reported antimycobacterial activity of major compounds identified in promising samples by GC–MS

Compound No.	Annotated Compound	Reported antimycobacterial activity (MIC, µg/mL)	Mycobacterial strain	Assay	Reference
6	6,7-Epoxyoctadecanoic acid methyl ester	No studies reporting antimycobacterial activity were found			
14	Hexadecanoic acid	100	Mtb H37Rv	MTT	(Esquivel-Ferriño et al. 2014)
		50	MDR isolate M-26	MTT	
		256	*M. aurum A+*	MTT	(Seidel and Taylor 2004)
		>256	*M. smegmatis*	MTT	(Seidel and Taylor 2004)
		No antimycobacterial activity detected	*M. smegmatis*	Direct bioautography	(Saravanakumar et al. 2008)
		No antimycobacterial activity detected	Mtb H37Rv	BACTEC 460 TB	
		No antimycobacterial activity detected	MDR Mtb	BACTEC 460 TB	
		512	*M. fortuitum* ATCC 6841	MTT	(Stavri et al. 2004)
		25	Mtb H37Rv	MABA	(Sandoval-Montemayor et al. 2012)
		50	Mtb isolate H10	MABA	
		50	Mtb isolate M15	MABA	
		50	Mtb isolate M26	MABA	
		100	MDR isolate NIRT-001	Broth microdilution assay	(Nirmal et al. 2023)

		Reduced isoniazin MIC from >100 to 0.0078	MDR isolate NIRT-001	Broth microdilution assay	
		100	MDR isolate NIRT-002	Broth microdilution assay	
		Reduced isoniazin MIC from 32 to 0.5	MDR isolate NIRT-002	Broth microdilution assay	
		800	Recombinant *M. smegmatis* mc²155	Broth microdilution assay	
		Reduced isoniazin MIC from 8 to 0.5	Recombinant *M. smegmatis* mc²155	Broth microdilution assay	
15	Pentadecanoic acid	256	*M. aurum A+*	MTT	(Seidel and Taylor 2004)
		>256	*M. smegmatis* ATCC 14468	MTT	
		>256	*M. fortuitum* ATCC 6841	MTT	
		>256	*M. abscessus* ATCC 19977	MTT	
		>256	*M. phlei* ATCC 11758	MTT	
20	1-nonene	No studies reporting antimycobacterial activity were found			
30	Heptadecane	No studies reporting antimycobacterial activity were found			
31	Acetylcitric acid, tributyl ester	No studies reporting antimycobacterial activity were found			
43	Hexadecanoic acid,2-hidroxy-1-(hidroxymethyl) ethyl ester	No studies reporting antimycobacterial activity were found			
52	Cholesterol	No studies reporting antimycobacterial activity were found			
54	Stigmasterol	100	Mtb H37Rv	REMA	(Suja et al. 2017)
		128 mg/mL	Mtb H37Rv	BACTEC 12B	(Saludes et al. 2002)

		62.5	Mtb isolate AC83 resistant to rifampicin	MABA	(Nkot et al. 2018)
		15.75	Mtb isolate AC45 resistant to isoniazid	MABA	
56	Stigmast-5-en-3β-ol[a]	25	Mtb H37Rv	REMA	(Suja et al. 2017)
		32	Mtb H37Rv	BACTEC 12B	(Saludes et al. 2002)
		>1000	H37Ra	MRA	(Morrison et al. 2016)

Abbreviations: MIC, minimum inhibitory concentration; MIQ, minimum inhibitory quantity; MDR, multidrug-resistant; MABA, Microplate Alamar Blue Assay; REMA, Resazurin Microtiter Assay; MRA, Microplate Resazurin Assay; BACTEC 460 TB, radiometric susceptibility assay.

References

Esquivel-Ferriño PC, Clemente-Soto AF, Ramírez-Cabriales MY, et al (2014) Volatile Constituents Identified in Hexane Extract of Citrus sinensis Peel and Anti-Mycobacterial Tuberculosis Activity of Some of its Constituents. J Mex Chem Soc 58:431–434

Morrison SA, Li H, Webster D, et al (2016) Antimycobacterial triterpenes from the Canadian medicinal plant *Sarracenia purpurea*. Journal of Ethnopharmacology 188:200–203. https://doi.org/10.1016/j.jep.2016.04.052

Nirmal CR, Rajadas SE, Balasubramanian M, et al (2023) Dodecanoic acid & palmitic acid disarms rifampicin resistance by putatively targeting mycobacterial efflux pump Rv1218c. Indian Journal of Medical Research 157:192–203. https://doi.org/10.4103/ijmr.ijmr_1610_22

Nkot JL, Ngono Bikobo DS, Abouem A Zintchem A, et al (2018) Antitubercular evaluation of root extract and isolated phytochemicals from Lophira lanceolata against two resistant strains of Mycobacterium tuberculosis. Pharm Biol 56:318–324. https://doi.org/10.1080/13880209.2018.1476559

Saludes JP, Garson MJ, Franzblau SG, Aguinaldo AM (2002) Antitubercular constituents from the hexane fraction of Morinda citrifolia Linn. (Rubiaceae). Phytotherapy Research 16:683–685. https://doi.org/10.1002/ptr.1003

Sandoval-Montemayor NE, García A, Elizondo-Treviño E, et al (2012) Chemical Composition of Hexane Extract of Citrus aurantifolia and Anti-Mycobacterium tuberculosis Activity of Some of Its Constituents. Molecules 17:11173–11184. https://doi.org/10.3390/molecules170911173

Saravanakumar DEM, Folb PI, Campbell BW, Smith P (2008) Antimycobacterial Activity of the Red Alga *Polysiphonia virgata*. Pharmaceutical Biology 46:254–260. https://doi.org/10.1080/13880200701739413

Seidel V, Taylor PW (2004) In vitro activity of extracts and constituents of *Pelagonium* against rapidly growing mycobacteria. International Journal of Antimicrobial Agents 23:613–619. https://doi.org/10.1016/j.ijantimicag.2003.11.008

Stavri M, Schneider R, O'Donnell G, et al (2004) The antimycobacterial components of hops (Humulus lupulus) and their dereplication. Phytotherapy Research 18:774–776. https://doi.org/10.1002/ptr.1527

Suja K, Jose L, Lakshmanan D, et al (2017) Isolation and Characterization of Antimycobacterial Compounds from Fruits of *Aegle marmelos* (L.) Correa. Journal of Communicable Diseases (P-ISSN: 0019-5138) 49:7. https://doi.org/10.24321/0019.5138.201732
